# U. S. V. M. A.

**Published:** 1896-01

**Authors:** 


### U. S. V. M. A.

*Committee on Army Legislation.*—Dr. J. P. Turner, Ft. Myer, Virginia; Dr. John R. Hart, 2577 Amber St., Philadelphia, Pa.; Dr. F. H. Mackie, Fair Hill, Maryland.

*Committee on Military Affairs of the Senate.*—Hawley (Chairman). Republicans: Proctor, Shoup, Sewell, Warren, Elkins. Democrats: Bates, Cockrell, Palmer, Mitchell (of Wisconsin), Walthall.

*Committee on Military Affairs of the House of Representatives.*—Hull, Iowa (Chairman); Curtis, New York; Marsh, Illinois; Woomer, Penna.; Griffin, Wisconsin; Southwick, New York; Parker, New Jersey; Bishop, Michigan; Fenton, Ohio; Catron, New Mexico; Tracy, Nebraska (Rep.); Tarsney, Missouri; Tyler, Virginia; McClellan, New York; Washington, Tennessee; Hart, Penna.; Lockhart, North Carolina (Dem.).

### BILL INTRODUCED BY REPRESENTATIVE DOVENOR, OF WEST VIRGINIA, IN THE HOUSE.

An Act to fix the pay, allowances, tenures of office, and rank of the Veterinary Surgeons of the United States Army.

Be it enacted by the Senate and House of Representatives of the United States of America, in Congress assembled:

Section 1. That the Veterinary Surgeon of the United States Army shall be given the pay, allowances, tenure of office, and relative rank of Second Lieutenants of Cavalry.

Sec. 2. That the number of Veterinary Surgeons in the Army of the United States shall not exceed two (2) for each regiment of Cavalry.

Sec. 3.[1] That hereafter all appointments as Veterinary Surgeons in the Army of the United States shall be confined to graduates of the recognized Veterinary Colleges of the United States, and candidates for such appointments shall be citizens of the United States and shall be required to pass such examination as the Secretary of War shall direct.

The Army Legislative Committee, Messrs. Turner, Hart, and Mackie, are all known as hard workers, and they have staked their reputations as sure winners in the battle for official recognition just opened. They will have as aids in their work this year the best organized and trained assistants that have ever supported such a Committee.

Buffalo had but one competitor in the battle for the meet-

[1] Added at suggestion of Chairman Hull of Military Committee of the House.

ing of the U. S. V. M. A. Nashville, Tennessee, was strongly pressed for this year's gathering, especially in view of the Centennial Anniversary of that city, at which time they will hold an exposition. The Southern veterinarians, alive to the value and influence of these annual gatherings to any community, and appreciating how much they raise the standard of the profession in new territory, made a strong plea for this recognition. We are sure that at no distant day the National Association will visit some one of the central Southern cities.

The Committee on Diseases, this year, with Dr. S. J. J. Harger as Chairman, will have as a western representative Dr. W. B. Niles, of Ames, Iowa, whose great interest and efficient efforts will always be remembered gratefully in connection with the Des Moines meeting.

The first eight votes cast for the place of meeting were all in favor of Buffalo.

Secretary Stewart has entered upon his work with a zeal and interest that will tell strongly upon the meeting of 1896.

We understand that the Chairman of the Publication Committee will have something of interest to say at Buffalo, and we are informed that he will say it strong.

Congressman Hull, of Iowa, whom many will remember with pleasure attended the banquet at the Des Moines meeting, and who won the good-will and fellowship of the Association members by his friendly remarks on Army Legislation, and who has been honored by Speaker Reed with the Chairmanship of the Committee on Military Affairs, has already given our Army Legislative Committee a friendly hearing and many valuable suggestions.

